# Omics-squared: human genomic, transcriptomic and phenotypic data for genetic analysis workshop 19

**DOI:** 10.1186/s12919-016-0008-y

**Published:** 2016-10-18

**Authors:** John Blangero, Tanya M. Teslovich, Xueling Sim, Marcio A. Almeida, Goo Jun, Thomas D. Dyer, Matthew Johnson, Juan M. Peralta, Alisa Manning, Andrew R. Wood, Christian Fuchsberger, Jack W. Kent, David A. Aguilar, Jennifer E. Below, Vidya S. Farook, Rector Arya, Sharon Fowler, Tom W. Blackwell, Sobha Puppala, Satish Kumar, David C. Glahn, Eric K. Moses, Joanne E. Curran, Farook Thameem, Christopher P. Jenkinson, Ralph A. DeFronzo, Donna M. Lehman, Craig Hanis, Goncalo Abecasis, Michael Boehnke, Harald Göring, Ravindranath Duggirala, Laura Almasy, The T2D-GENES Consortium

**Affiliations:** 1 South Texas Diabetes and Obesity Institute, University of Texas Rio Grande Valley, Harlingen, TX 78550 USA; 2 Department of Biostatistics, Center for Statistical Genetics, University of Michigan, Ann Arbor, MI 48109 USA; 3 Department of Epidemiology, Human Genetics and Environmenal Sciences, University of Texas Health Science Center at Houston, Houston, TX 77030 USA; 4 Department of Genetics, Massachusetts General Hospital, Boston, MA 02114 USA; 5 Genetics of Complex Traits, Peninsula College of Medicine and Dentistry, University of Exeter, Exeter, UK; 6 Department of Genetics, Texas Biomedical Research Institute, 7620 NW Loop 410, San Antonio, TX 78227 USA; 7 Cardiovascular Division, Baylor College of Medicine, Houston, TX 77030 USA; 8 Division of Clinical Epidemiology, Department of Medicine, University of San Antonio Health Science Center at San Antonio, San Antonio, TX 78229 USA; 9 Department of Psychiatry, Yale University, New Haven, CT 06106 USA; 10 Centre for Genetic Origins of Health and Disease, University of Western Australia, Crawley, Australia; 11 Department of Biochemistry, Faculty of Medicine, Kuwait University, Safat, Kuwait City, 13110 Kuwait; 12 Texas Diabetes Institute, University of San Antonio Health Science Center at San Antonio, San Antonio, TX 78229 USA; 13 Department of Genetics, University of Pennsylvania, Philadelphia, PA 19104 USA

## Abstract

**Background:**

The Genetic Analysis Workshops (GAW) are a forum for development, testing, and comparison of statistical genetic methods and software. Each contribution to the workshop includes an application to a specified data set. Here we describe the data distributed for GAW19, which focused on analysis of human genomic and transcriptomic data.

**Methods:**

GAW19 data were donated by the T2D-GENES Consortium and the San Antonio Family Heart Study and included whole genome and exome sequences for odd-numbered autosomes, measures of gene expression, systolic and diastolic blood pressures, and related covariates in two Mexican American samples. These two samples were a collection of 20 large families with whole genome sequence and transcriptomic data and a set of 1943 unrelated individuals with exome sequence. For each sample, simulated phenotypes were constructed based on the real sequence data. ‘Functional’ genes and variants for the simulations were chosen based on observed correlations between gene expression and blood pressure. The simulations focused primarily on additive genetic models but also included a genotype-by-medication interaction. A total of 245 genes were designated as ‘functional’ in the simulations with a few genes of large effect and most genes explaining < 1 % of the trait variation. An additional phenotype, Q1, was simulated to be correlated among related individuals, based on theoretical or empirical kinship matrices, but was not associated with any sequence variants. Two hundred replicates of the phenotypes were simulated. The GAW19 data are an expansion of the data used at GAW18, which included the family-based whole genome sequence, blood pressure, and simulated phenotypes, but not the gene expression data or the set of 1943 unrelated individuals with exome sequence.

## Background

Genetic Analysis Workshop 19 (GAW19) concentrated on approaches to identify and characterize loci and genetic variants influencing quantitative and complex phenotypes through analysis of genome sequence and gene expression levels. Data distributed for the workshop included whole genome sequence (WGS) and gene expression in 959 individuals in a set of 20 Mexican American families [1] and whole exome sequence in a set of 1943 unrelated Mexican American individuals drawn from a larger multi-ethnic case/control study [2]. The genotype calls provided for odd-numbered autosomes previously underwent quality control screening and were pre-cleaned. Both real and simulated phenotype data were provided. The real phenotypes concentrated on systolic and diastolic blood pressure and related covariates, including age, year of examination, use of antihypertensive medications, and tobacco smoking. Simulated phenotypes were designed to mimic various aspects of these real phenotypes, including the distribution of the quantitative traits, overall heritabilities, and correlations between traits. Both real and simulated phenotypic data from the family sample were longitudinal, with up to four time points in the real data and three time points in the simulated data. The unrelated data set was cross- sectional, with a single time point available in both the real and simulated data sets.

## Methods

### Whole genome sequence in families

The family data set distributed for GAW19 is an expanded version of that used for GAW18, which has been previously described in detail [1]. We provide here only a brief summary of this data set. The core of the family data set was WGS data for 464 individuals in 20 large Mexican American families drawn from Type 2 Diabetes Genetic Exploration by Next-generation sequencing in multi-Ethnic Samples (T2D-GENES) Project 2, and measures of systolic and diastolic blood pressure. Called WGS variants were available for 464 key individuals. These individuals were selected to provide comprehensive data on all alleles present in a pedigree and their phase, with two parents and one child per sibship sequenced when possible or multiple children sequenced when one or more parents was unavailable. Genotypes were imputed for the remaining 495 family members based on a genome-wide association study (GWAS) framework of dense SNPs genotyped in all family members. Directly typed or imputed WGS data for odd numbered autosomes were provided for 959 individuals in these 20 families. Systolic and diastolic blood pressures were measured at up to four time points over a span of 20 years, and were available for 932 of the 959 family members. Three or more measures were available for 503 individuals (52.5 %) and 686 individuals (73 %) had at least two measurements. Hypertension was defined as systolic blood pressure (SBP) > 140, diastolic blood pressure (DBP) > 90, and/or use of antihypertensive medications at that examination. The prevalence of hypertension varied from 18 % at the first exam to 52 % at the fourth exam as the cohort aged. Accompanying covariate data included sex, age at examination, year of examination, current use of antihypertensive medication, and current tobacco use. Year of examination was provided to allow for examination of temporal trends as the examinations spanned a 20-year period.

### Gene expression in the family data set

For GAW19, the GAW18 family data set was expanded with the addition of genome-wide gene expression measures in a subset of the T2D-GENES WGS families drawn from the San Antonio Family Heart Study (SAFHS). Measures of gene expression were generated using version 1 of Illumina Sentrix Human Whole Genome (WG-6) microarrays containing 47,293 probes in total. The SAFHS transcript data set is described in Gӧring et al. [3] and details of laboratory procedures can be found there. However, the GAW19 data set was constructed using a somewhat different analytical processing pipeline than that described previously.

Briefly, gene expression data were generated from peripheral blood mononuclear cells (PBMCs) from 1,371 samples in total (including controls of various types and duplicate samples). Based on the per-sample number of probes with significantly detectable expression level (counts of reported “detection *p*-values” of less than or equal to 0.05 across all probes), the mean raw expression level (reported “average signals”) across all probes, and the mean correlation of any sample against all other samples (in the reported “average signals” of all probes), 1244 unique samples (out of 1280 in total) were identified as yielding expression data of adequate quality and were kept for further processing. Of these 1244 high quality samples, 647 come from individuals in the 20 T2D-GENES WGS families and were included in the GAW19 family data set.

Among these samples, we tested separately for each probe whether there was significant detectable expression, by conducting a binomial test based on counts of samples with and without reported “detection *p*-values” of less than or equal to 0.05. Subsequently, we calculated the false discovery rate (FDR) across all probes. A total of 20634 transcripts were significant at a FDR of 0.05 and were kept for further processing. Subsequently, we shifted all “average signals” upwards so that the observed minimum value (in any probe in any sample) was 1.0, conducted a log2 transformation followed by a quantile normalization transformation. The resulting data were distributed for GAW19.

### Exome data in unrelated individuals

The second data set distributed for GAW19 was a large sample of unrelated Mexican American individuals drawn from T2D-GENES Project 1 [2]. Overall, this project included 10,000 whole exome sequences from five ancestry groups, with approximately 1000 cases with T2D and 1000 controls from each group. This project was designed to study the role of uncommon variation and to identify potential functional variants behind previously identified GWAS signals.

The GAW19 exome data set included 1,943 Hispanic samples whole‐exome sequenced as part of T2D‐GENES Project 1. These samples were drawn from five separate family-based studies, the San Antonio Family Heart Study [4], the San Antonio Family Diabetes/Gallbladder Study [5], the Veterans Administration Genetic Epidemiology Study [6], the Family Investigation of Nephropathy and Diabetes study family component [7], and a study from Starr County, Texas [8, 9]. Approximately 75 % of the sample of unrelated individuals came from the Starr County study. The 1943 individuals include 1021 with T2D and 922 non-diabetic controls. Information on T2D diagnosis was not provided as part of the GAW19 data set.

Phenotypic data provided included year of examination, SBP, DBP, and use of anti-hypertension medication; however, some of these variables were only available from a subset of the studies in the data set. Year of examination was available for 409 individuals and ranged from 1991 to 2012. Use of anti-hypertensive medications was available for 407 individuals, of whom 147 were on such medications and 260 were not. A total of 1851 individuals had measurements of systolic and diastolic blood pressure. SBP ranged from 66–213, with a mean of 125. DBP ranged from 32–123, with a mean of 73.5. These values are similar to those in the GAW19 family data set, where mean SBP was 122–128 across the four examinations and mean DBP was 71–78. Data on nicotine use, provided for the GAW19 family sample, were not available for the exome sample.

Exomic regions were isolated using Agilent Truseq capture reagents, and individually‐barcoded samples were sequenced on Illumina HiSeq2000 instruments. Across the coding sequence of 18,281 genes the average read depth was 81.7‐fold. Sequence reads were processed and aligned to the reference genome (hg19) with Picard (http://broadinstitute.github.io/picard/). Polymorphic sites and genotypes were called with GATK [10].

Samples and variants were excluded on the basis of multiple quality control metrics: array genotype concordance (where available), mean heterozygosity and homozygosity, high singleton counts for samples, Variant Quality Score Recalibration (VSQR) for single nucleotide variants (SNVs), and hard filtering for small insertion-deletion variants (INDELs). Within each ethnicity in the overall T2D-GENES Project 1 data set, variants were excluded on the basis of call rate (<90 % in any study in ancestry group), deviation from Hardy‐Weinberg equilibrium (exact *p* < 10^−6^ in any study in ancestry group) or differential call rate between T2D cases and controls (*p* < 10^−4^ in all studies combined across ancestry group). Autosomal variants that passed extended QC and with MAF > 1 % in all ancestry groups were used for trans‐ethnic kinship analyses. Identity‐by‐state (IBS) sharing between each pair of samples was calculated on the basis of independent variants (trans‐ethnic r2 < 0.05) and axes of genetic variation were constructed through principal components analysis implemented in EIGENSTRAT [11] to identify ethnic outliers. Only individuals in the Mexican American subset of T2D-GENES Project 1 were included in the GAW19 exome data set.

For GAW19, variant call format (VCF) files were provided for odd‐numbered autosomes. These included genotype calls in the NALTT field, which contained only high quality (GQ > 20) genotypes scored as 0/1/2, genotypes subjected to only minimal quality control in the GT field, and likeihood-based estimates of allele dosages in the DOS field. These VCF files included 1,689,048 SNVs (some of which were multiallelic) and 76,397 INDEL variants. Because the Mexican American exome data set provided for GAW19 was drawn from T2D-GENES’ larger multi-ethnic case/control sample, some markers included in the GAW19 exome data were monomorphic as they varied in the overall T2D-GENES sample but not in the Mexican American subset. Considering only SNVs with at least five observed copies of the minor allele, which might be individually analyzed, Table 1 shows the distribution of these variants across annotation categories in the unrelated exome sample and in the family WGS sample. In general, although the family sample had fewer individuals sequenced, there were more variants present in five or more copies in each annotation category. Some general patterns are similar across the unrelated exome and family WGS data sets, with 51 % of coding variants in each case being non-synonymous and with the proportion of SNVs with minor alleles frequencies < = 1 % increasing when moving from coding variants to non-synonymous variants to variants rated as highly deleterious by PolyPhen-2 [12].

**Table 1:** Variant annotation, by minor allele frequency category, for variants present in at least 5 copies in the unrelated exome sample and in the family WGS sample

Sample	Variant type		0.1 < MAF^a^ ≤ 0.5	0.01 < MAF ≤ 0.1	MAF ≤ 0.01
Unrelated exome sample	Coding	All coding	5956	6878	13776
		Synonymous	3240	3329	6159
		Non-synonymous	2647	3450	7389
		Highly Deleterious	340	833	1979
	Non-coding	5′ UTR^b^	165	170	361
		3′ UTR	309	335	588
Family WGS sample	Coding	All coding	15270	16412	23872
		Synonymous	8089	7743	10061
		Non-synonymous	6828	8275	13180
		Highly Deleterious	549	1159	1996
	Non-coding	5′ UTR	3142	3057	4093
		3′ UTR	16628	16688	21885

^a^ *MAF* minor allele frequency. ^b^ *UTR* untranslated region

Although the exome sample was intended to be unrelated individuals, analysis of the SNV-based kinship estimates among individuals in the GAW19 unrelated data set shows that there are a few relative pairs present in the sample. While most of these relationships are third degree or more distant, a few first- or second-degree relative pairs are present in the GAW19 exome sample.

### Simulated phenotypes

A set of simulated phenotypes was constructed using the same model in both the exome and family data sets to closely match the observed phenotypic data. Simulated SBP and DBP had the same mean, variance, heritability and correlations with each other as observed in the real data. The observed data were also used to model covariate effects, with blood pressures being higher in males than females and increasing with age. A total of 200 replicates of the simulated phenotypes were generated. For the WGS family data set, phenotypes were simulated longitudinally, at three time points at 5-year intervals. Genetic parameters remained the same across all three exams and random environmental components were given a correlational structure based on that seen in the real data. In the exome data set of unrelated individuals, simulated SBP and DBP were modeled for a single time point, with the same mean, variance, age, sex, and medication effects as in the family sample. The age and sex of each individual were drawn from the real data and did not vary across the 200 simulation replicates that were generated.

‘Functional’ genes for the simulation were selected based on correlations of gene expression with measures of SBP and DBP in the SAFHS. To meet inclusion criteria, a gene’s expression levels in the SAFHS had to be both phenotypically and genetically correlated with observed SAFHS SBP or DBP. Within these selected genes, non-coding variants within 5 kb upstream and downstream of the gene that were associated with expression levels of that gene were declared ‘functional’ for the simulations as were coding variants predicted by PolyPhen-2 [12] to be deleterious. Effect sizes in the simulation for each SNP were determined using the observed correlation between mRNA levels and blood pressure in the SAFHS for the non-coding variants and using a function of PolyPhen-2 score (PP2S) for the coding variants:

$$ \left(\mathrm{percentile}\ \mathrm{of}\ \mathrm{ranked}\ \mathrm{P}\mathrm{P}2\mathrm{S}\right) \times \left(\mathrm{P}\mathrm{P}2{\mathrm{S}}^2\right) \times \kern0.5em {\uprho}_g\kern0.5em \times \kern0.5em k\kern0.5em \times \kern0.5em l $$

where ρ_g_ is the genetic correlation between mRNA levels and SBP or DBP, *k* is an overall constant, and *l* is a gene-specific constant.

There were 245 genes selected to influence simulated SBP and/or DBP. In the family data set, these 245 genes contained 1458 functional variants whose effect sizes ranged from < 0.001 to 2.78 % of the total phenotypic variance. The gene with the largest effect, *MAP4* on chromosome 3, accounted for 7.79 % of phenotypic variance in simulated SBP and 6.48 % in simulated DBP when effects of all ‘functional’ variants within and flanking the gene were combined. A list of the functional variants with the largest effect sizes in the family data set is available in the GAW18 data description [1].

The variants designated as ‘functional’ for phenotype simulations in the GAW19 exome data set differ slightly from those in the family data set, due to non-coding variants present in the WGS but not covered by the exome sequencing and due to new coding variants present in the larger set of unrelated individuals in the exome data set that had not been represented in the smaller family data set. The 245 ‘functional’ genes used in simulating phenotypes for the family data set were screened for new non-synonymous coding variants present in the unrelated exome data set and new variants were assigned effect sizes by the same formula, as a function of their PolyPhen-2 scores. This resulted in a total of 1730 functional variants in the exome data set. The 20 variants with the largest effect sizes in the exome sample are shown in Table 2. All are non-synonymous coding variants. Effect sizes were somewhat larger for the GAW19 exome simulation than for the simulations in the family data set. The *MAP4* variant with the largest effect in the families, is only the fourth largest effect in the unrelated exome simulation.

**Table 2:** Top 20 variants influencing simulated SBP and DBP in the GAW20 unrelated exome sample, in decreasing order of variance explained

Chromosome	Position (bp)	Gene	Frequency of non-reference allele	Beta^a^ DBP	Beta^a^ SBP	DBP variance explained (%)	SBP variance explained (%)
3	47956424	MAP4	0.34354	−3.93	−6.09	7.88	7.01
1	175092674	TNN	0.64771	3.38	4.10	5.89	3.20
1	66075952	LEPR	0.15466	3.49	3.78	3.61	1.56
3	48040283	MAP4	0.02805	−5.03	−7.80	1.56	1.39
3	47957996	MAP4	0.02290	−4.41	−6.84	0.98	0.87
1	151501841	CGN	0.10396	−2.63	0	1.46	0
13	28624294	FLT3	0.61400	1.40	1.44	1.05	0.41
3	48040284	MAP4	0.00695	−5.05	−7.83	0.40	0.36
1	175092637	TNN	0.02007	3.38	4.10	0.51	0.28
1	151491026	CGN	0.02084	−2.88	0	0.38	0
1	175046835	TNN	0.01390	2.88	3.49	0.26	0.14
3	47908815	MAP4	0.00257	−4.99	−7.73	0.15	0.13
11	77937768	GAB2	0.00386	0	6.04	0	0.12
1	175046652	TNN	0.00824	3.35	4.06	0.20	0.11
9	123605126	PSMD5	0.18837	−0.58	−0.89	0.11	0.10
3	58109162	FLNB	0.45677	0.13	0.64	0.01	0.08
1	151503071	CGN	0.01132	−2.88	0	0.21	0
1	175054626	TNN	0.00566	3.30	4.00	0.14	0.08
1	53712727	LRP8	0.21410	0	−0.71	0	0.07
19	46812451	HIF3A	0.03886	1.36	0.92	0.16	0.03

^a^Beta = change in mean phenotype value per non-reference allele carried

In simulations in the family data set, frequency of simulated anithyptertensive medication usage was modeled on the observed data. Simulated SBP and DBP were each reduced by treatment, except in individuals carrying deleterious variants in the CYP3A43 gene, producing a genotype-by-medication interaction effect. In the exome data set, because only a subset of individuals had information available on medication usage, medication status was randomly assigned, varying across replicates, and was based on the proportion of participants on antihypertensive medications in exam 1 in the family sample.

In addition to the measured genetic effects generated from the sequence data, an aggregate, unspecified additive genetic residual was modeled based on pedigree-derived kinship estimates in the family data set and on empirical kinship estimates among all pairs of individuals estimated using the program LDAK [13] in the exome data set. This residual additive genetic correlation was set to maintain the heritabilities of simulated SBP and DBP and the genetic correlations between them as observed in exam 1 of the family data set.

Pedigree-derived and empirical kinship estimates also were used to simulate a phenotype called Q1 that had a heritability of 68 % but was independent of the WGS or exome sequence variants. Q1 was simulated as a normally distributed quantitative trait and was designed primarily for testing of type I error. Only a single time point was simulated for 200 replicates of Q1 in both the family and exome data sets.

## Conclusions

The GAW19 data provide a broad range of analytical possibilities, including both genomic and transcriptomic data; both family and unrelated cohort data sets; both real and simulated phenotypes; and both longitudinal and cross sectional data sets. At the workshop, investigators used these data to address a wide variety of topics. Analytical issues addressed included methods for population- [14] and family-based [15] association, machine learning and data mining approaches to gene localization [16], and methods for joint analysis of mutiple phenotypes [17]. Some groups concentrated on approaches to dealing with multiple testing in these high dimensional sequence data by filtering sequence variants or placing informative priors for association analyses [18], by pathway-based approaches for gene localization [19], or by other variant collapsing approaches [20]. Other contributions focused on utilizing unique aspects of the GAW19 family data set, including genetic analyses of longitudinal data [21], and analysis of gene expression data [22]. The variety of topics addressed in these GAW19 contributions illustrate the utility and versatility of the GAW19 data. As many genetic studies of complex human phenotypes are currently focusing on exome and whole genome sequence, and their integration with gene expression data, we anticipate that the GAW19 data will continue to provide a rich resource for statistical genetic methods development, comparison, and testing for years to come.
